# Proliferation, differentiation and amyloid-β production in neural progenitor cells isolated from TgCRND8 mice

**DOI:** 10.1016/j.neuroscience.2013.12.021

**Published:** 2014-03-07

**Authors:** S. Kanemoto, J. Griffin, K. Markham-Coultes, I. Aubert, A. Tandon, P.S. George-Hyslop, P.E. Fraser

**Affiliations:** a Tanz Centre for Research in Neurodegenerative Diseases, University of Toronto, 60 Leonard Avenue, Toronto, ON M5T 2S8, Canada; b Sunnybrook Research Institute and Department of Laboratory Medicine and Pathobiology, University of Toronto, 2075 Bayview Avenue, Toronto, ON M4N 3M5, Canada; c Department of Medicine (Neurology), University of Toronto, Canada; d Cambridge Institute for Medical Research, Cambridge National Institute of Health Research, Biomedical Research Unit in Dementia, University of Cambridge, Cambridge CB2 0XY, UK; e Department of Medical Biophysics, University of Toronto, Canada

**Keywords:** AD, Alzheimer’s disease, AICD, amyloid precursor protein intracellular domain, ANOVA, analysis of variance, APP, amyloid precursor protein, Aβ, amyloid-β, βFGF, fibroblast growth factor, BrdU, 5-bromo-2-deoxyuridine, DMEM, Dulbecco's modified Eagle medium, DMSO, dimethyl sulfoxide, EGF, epidermal growth factor, ELISA, enzyme-linked immunoabsorbent assay, FBS, fetal bovine serum, GFAP, glial fibrillary acidic protein, HRP, horse radish peroxidase, NeuN, neuronal nuclear antigen, Non-Tg, non-transgenic, NPCs, neural progenitor cells, PBS, phosphate-buffered saline, PI, propidium iodide, S.D., standard deviation, SGZ, subgranular zone, SVZ, subventricular zone, neural progenitor cells, amyloid, Alzheimer’s disease, transgenic mice

## Abstract

The amyloid precursor protein (APP) and amyloid-β (Aβ) peptide play central roles in the pathology and etiology of Alzheimer’s disease. Amyloid-induced impairments in neurogenesis have been investigated in several transgenic mouse models but the mechanism of action remains to be conclusively demonstrated. The changes in neurogenesis during this transition of increasing Aβ levels and plaque formation were investigated in the present study. We found that the proliferation of newborn cell in the dentate gyrus was enhanced prior to elevations in soluble Aβ production as well as amyloid deposition in 5-week-old TgCRND8 mice, which are well-established Alzheimer’s disease models, compared to non-transgenic (Non-Tg) mice. The number of BrdU-positive cells remained higher in TgCRND8 vs Non-Tg mice for a period of 8 weeks. The numbers of BrdU/NeuN-positive cells were not significantly different in TgCRND8 compared to Non-Tg mice. A significant decrease in BrdU/GFAP but not in BrdU/S100β was found in Tg vs Non-Tg at 6-weeks of age. In addition, a unique observation was made using isolated neuroprogenitor cells from TgCRND8 mice which were found to be less viable in culture and produced substantial amounts of secreted Aβ peptides. This suggests that the proliferation of neural progenitors *in vivo* may be modulated by high levels of APP expression and the resulting Aβ generated directly by the progenitor cells. These findings indicate that cell proliferation is increased prior to Aβ deposition and that cell viability is decreased in TgCRND8 mice over time.

## Highlights

• In young TgCRND8 mice, proliferation of newborn cells in dentate gyrus is increased, compared with Non-Tg control mice.
• On the contrary, differentiation to neural progenitor cells of newborn cells in the TgCRND8 hippocampus is impaired.
• Neurosphere cultures isolated from hippocampi of TgCRND8 mice show low viability than those from Non-Tg control.
• Neurosphere cultures from TgCRND8 mice secrete high levels of Aβ peptide.

## Introduction

Neurogenesis occurs throughout life with active adult neurogenesis occurring in the subventricular zone (SVZ) in the lateral ventricle and the subgranular zone (SGZ) of the dentate gyrus in the hippocampus (Gage, 2000). Neurogenesis in SVZ is related to odor discrimination and memory (Gheusi et al., 2000; Rochefort et al., 2002; Magavi et al., 2005). In the SGZ new neurons are involved in learning and memory (Deng et al., 2009). During neurogenesis, various key factors contribute to the process of differentiation from neural stem cells to mature neurons (Ming and Song, 2011). For example, glial fibrillary acidic protein (GFAP) is expressed in the first stage of neurogenesis. Meanwhile, the neuronal nuclear antigen (NeuN) is induced in the later phases.

Alzheimer’s disease (AD) is a prevalent neurodegenerative disorder, the principle clinical feature of which is progressive dementia beginning in late adult life. This is accompanied by a characteristic set of neuropathological features including senile plaques, neurofibrillary tangles, and neurodegeneration of specific areas of the brain. The hippocampal formation is one of the most affected areas in the AD brain (Selkoe, 2000). Senile plaques are composed of extracellular accumulation of amyloid-β (Aβ) peptides. Neurofibrillary tangles are intracellular aggregates of hyper-phosphorylated tau proteins, which function to stabilize neuronal microtubules under normal conditions. At present three causative genes are known to be associated with autosomal dominant, fully penetrant familial AD, namely, amyloid precursor protein (APP), presenilin-1 (PS1) and presenilin-2 (PS2) (Goate et al., 1991; Sherrington et al., 1995; Rogaev et al., 1995). Mutations in these proteins lead to alterations in the processing of Aβ peptides from APP, resulting in more toxic forms of Aβ peptides in plaques. Several animal models of AD pathology related to the three genes have been established. To date, many studies using the animal models of AD have been performed to elucidate the correlation between AD pathology and neurogenesis (Lazarov and Marr, 2010; Mu and Gage, 2011). There are a few studies that report understanding of neurogenesis on AD (Jin et al., 2004; Ziabreva et al., 2006), but the status of neurogenesis individuals with AD has not been defined yet.

In this study, we investigated the effect of AD pathology on neurogenesis using well-established TgCRND8 mouse model of AD (Chishti et al., 2001). In TgCRND8 mice, prior to the onset of increased soluble Aβ peptide production and amyloid plaque accumulation, the number of newborn cells in the SGZ was greater than that of non-transgenic (Non-Tg) littermates. However, once the brain levels of Aβ began to increase, the number of newborn cells fell, and their differentiation to neural progenitor cells (NPCs) was impaired in TgCRND8 compared to that of Non-Tg littermates. These results indicate that at later stages where Aβ and APP are increased there is a decrease in the number of progenitor cells and their subsequent differentiation in TgCRND8 mice is impaired. The investigation of neurospheres isolated from the amyloid mice displayed a significant decrease in cell viability that may be related, in part, to Aβ toxicity.

## Experimental procedures

### Transgenic mouse model

TgCRND8 mice expressing both human Swedish mutant and Indiana mutant of APP, driven by the hamster prion (PrP) promoter were previously characterized (Chishti et al., 2001). Animals at different stages of development were investigated to determine the effects of amyloid pathology on neurogenesis. TgCRND8 mice and Non-Tg littermates at 5 weeks of age were injected with 5-bromo-2-deoxyuridine (BrdU) intraperitoneally twice a day for 5 days. Animals were sampled at intervals of 1, 2, 4, 6 or 8 weeks from the start date of injection. All animal studies followed the University of Toronto Animal Care Policies and Guidelines and were approved by the University Animal Care Committee of University of Toronto.

### Antibodies

The following antibodies were used for this study. Rat anti-BrdU monoclonal (1:2000; Accurate Chemical & Scientific), mouse anti-NeuN monoclonal (1:2000; Chemicon) for mature neuron, mouse anti-S100β monoclonal (1:2000; Sigma–Aldrich) for mature astrocytes, and mouse anti-GFAP monoclonal (1:2000; Cell Signaling) for neural precursor and mature astrocytes. As secondary antibodies, donkey anti-rat conjugated with horse radish peroxidase (HRP) and donkey anti-mouse conjugated with HRP (1:200; Jackson Immunoresearch Laboratory) were used.

### BrdU injection

BrdU was dissolved in phosphate-buffered saline (PBS) at the concentration of 3 mg/ml. BrdU solution was administered to mice intraperitoneally at the concentration of 50 mg/kg twice a day for five consecutive days.

### Immunohistochemistry

Immunohistochemistry was performed as described previously (Kee et al., 2007). Briefly, mice were perfused with 0.1 M PBS and with 4% buffered paraformaldehyde. Brains were removed from the skull and immersed in 4% paraformaldehyde at 4 °C for 12 h. Fixed tissues were placed in 30% sucrose at 4 °C until they settled at the bottom. Samples were frozen and sectioned at 50 μm with a cryostat. Sectioned samples were placed into 24-well plates filled with 0.1 M PBS (pH 7.4), washed with 0.1 M PBS three times, immersed into 1 N HCl for 30 min at 37 °C in order to denature DNA, and then placed in a blocking solution composed of mouse immunoglobulin G (1:200, Jackson Immunoresearch Laboratory) in 0.1 M PBS for 1 h at room temperature. Sections were then immersed into 1% hydrogen peroxide solution for 30 min at room temperature for quenching endogenous peroxidase, and incubated with primary antibodies at 4 °C for ∼15 h. Thereafter, sections were incubated with secondary antibodies for 1 h at room temperature. For signal enhancement, TSA-biotin and TSA-fluorescein system (Perkin Elmer) were used according to the manufacturer’s protocols. Following each step described above, 0.1 M PBS (pH 7.4) wash was conducted three times before proceeding to the next step. Nuclei were counter-stained with Hoechst 33258 (Sigma). After staining, sections were mounted on slides with antifade fluorescence mounting medium (Dako). Slides were observed with fluorescence microscopy or confocal microscopy, and BrdU-positive cells in the dentate gyrus were counted. A total of 48 sections from the dentate gyrus region were prepared from each animal with five animals in each condition were examined for the quantification. Sections were sorted sequentially to four groups (12 sections/group) and immunostained with BrdU only or BrdU plus the desired markers NeuN, S100β or GFAP. The total number of positive cells was counted from each of the entire sections.

### Neurosphere isolation

Neural stem cells were isolated from TgCRND8 mice and Non-Tg littermates at 4 months of age. The protocol of isolation was as previously described (Visanji et al., 2011). Briefly, whole brains of TgCRND8 mice and Non-Tg littermate mice (*n* = 10) were cut into pieces (∼2 mm^3^) and incubated at 37 °C in 10 ml/g tissue of Dulbecco's modified Eagle medium (DMEM) containing papain (2.5 units/ml), protease type 1 (1 unit/ml), and DNase I (250 units/ml). The tissue was triturated every 5 min until a smooth consistency was achieved (∼30 min). Following digestion, the cell suspension was mixed with an equal volume of DMEM/F-12/N2 containing 10% fetal bovine serum (FBS). The suspension was then passed through a sterilized 70-μm nylon mesh filter and centrifuged (1000×*g* for 3 min). The pellet was resuspended in DMEM/F-12/N2 containing 10% FBS and mixed with an equal volume of Percoll (9:1 v/v Percoll/PBS) and centrifuged (20,000×*g* for 30 min at 18 °C). NPCs were harvested from the low buoyancy fraction just above the red blood cell layer. Cells were washed in cold PBS containing an antibiotic–antimycotic solution (containing penicillin, streptomycin, and amphotericin B) before plating in DMEM/F-12/N2 containing and antibiotic–antimycotic solution, fibroblast growth factor-basic (βFGF) (20 ng/ml), epidermal growth factor (EGF) (20 ng/ml), and heparin (5 μg/ml). Proliferating NPC began to grow in clusters ∼2–3 weeks post-isolation. During this period, half the media was changed weekly, and βFGF (20 ng/ml), EGF (20 ng/ml), and heparin (5 μg/ml) were added every 3–4 days.

### Aβ quantification and γ-secretase inhibition

Aβ40 and Aβ42 levels were measured by enzyme-linked immunoabsorbent assay (ELISA) using conditioned medium collected from cultured neurospheres isolated from Non-Tg and TgCRND8 mice. The ELISA (Biosource International) was performed as previously described (Pardossi-Piquard et al., 2009). For Aβ quantification, neurosphere media was conditioned for 4 days with samples taken from paired cultures (Non-Tg and TgCRND8) between passages 3 and 7. To assess the effects of γ-secretase inhibition, cells were treated overnight with 100 nM Compound E (Santa Cruz) and dimethyl sulfoxide (DMSO) was used as a negative control as previously described (Beher et al., 2001).

### Propidium iodide and nuclear staining

Neurospheres were cultured in fresh DMEM/F12 growth medium supplemented with N2 (1X); Heparin 5 μg/ml; EGF 20 ng/ml; βFGF 20 ng/mL were plated in 12-well dishes (approximately 10,000 cells/well) (NUNC) and allowed to grow for 4 days undisturbed. At this point, the medium was carefully removed to avoid disturbing the cells and the cells were stained with propidium iodide (100 μg/ml) and Hoechst 33258 (50 μg/ml) for 15 min. The staining solution was removed and the cells were rinsed once with PBS pH 7.4. The cells were fixed in cold 4% buffered paraformaldehyde for 30 min. After fixation, the cells were rinsed three times with PBS pH 7.4 and stored in PBS in the dark until used. Hoechst and propidium iodide (PI)-positive cells were counted in six fields for Non-Tg and TgCRND8 NPCs. The percent dead (ratio of PI-positive/Hoechst-positive cells) was calculated for each field and compared using a Mann–Whitney *U*-test using the Prism statistical analysis software.

### Statistical analysis

Data are expressed as means ± standard deviation (S.D.) and statistical comparisons were performed with the two-way analysis of variance (ANOVA) or the Mann–Whitney *U*-test. Values that were statistically significant (*p* < 0.0001, *p* < 0.01 or *p* < 0.05) are indicated with an asterisk. Numbers of animals examined and replicates are provided in the appropriate figure legends.

## Results

The TgCRND8 transgenic mouse line is an aggressive model of Alzheimer-related amyloid pathology that results in widespread plaque deposition in the cortex and hippocampus at 4 months of age. Although APP expression in the transgenics is high post-natal, the levels of both Aβ40 and Aβ42 are only modestly elevated in the transgenics after birth (30–50 ng/g) as compared to Non-Tgs until about 8 weeks of age. However, at ∼9–10 weeks of age both Aβ40 and Aβ42 increase dramatically in the TgCRND8 mice (Chishti et al., 2001). At this stage, Aβ42 is elevated more than 10-fold and predominates over the Aβ40 species leading to a significantly increased Aβ42/40 ratio which results in a rapid acceleration of the amyloid accumulation and related neuropathological changes. In the present study, we investigated the changes in neurogenesis during the transition from low Aβ levels to increased soluble, possibly oligomeric forms, and the early stages of plaque formation.

### Proliferation of hippocampal newborn cells is increased prior to amyloid deposition

**Fig. 1:**
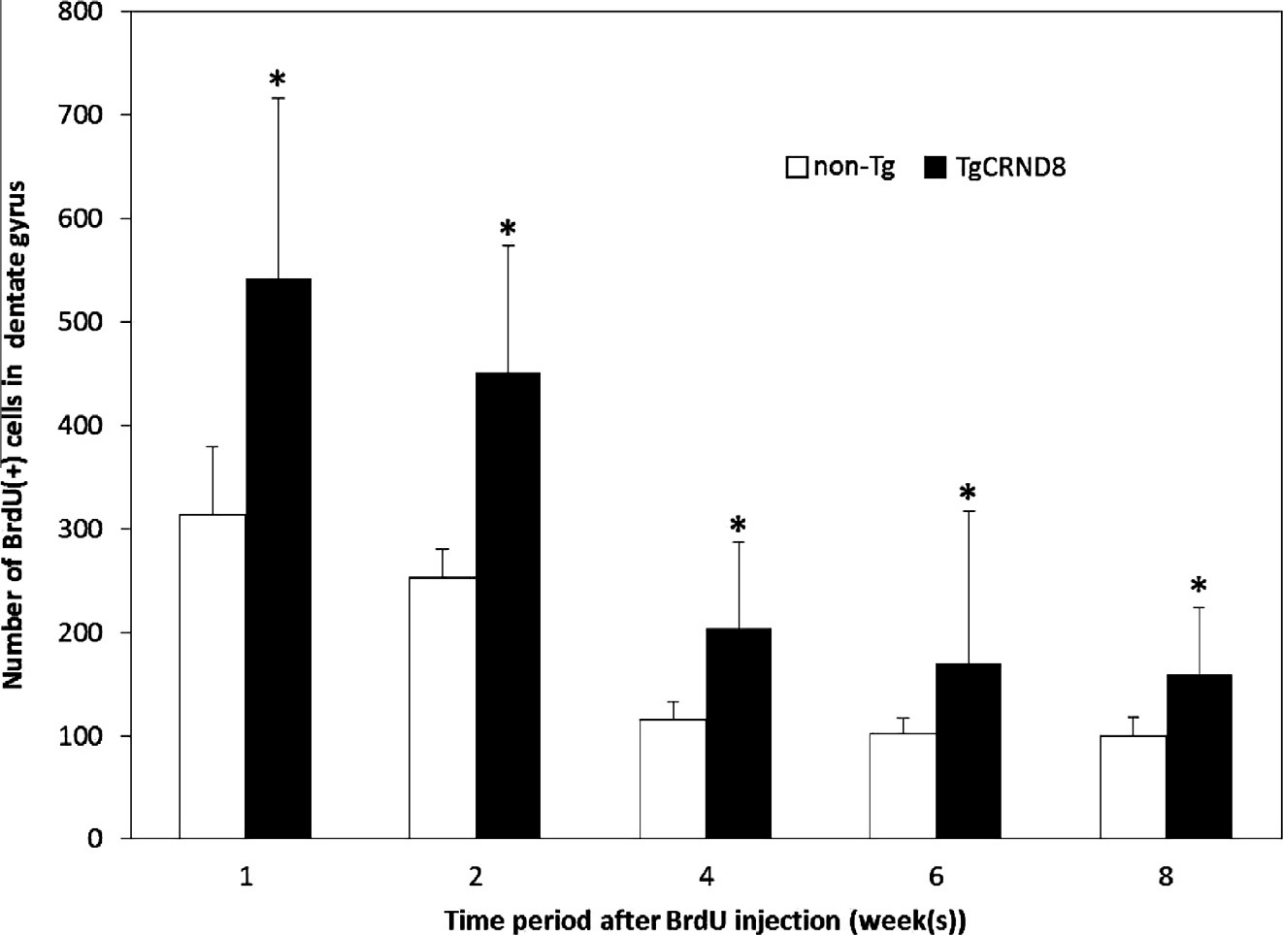
Cell proliferation in Non-Tg and TgCRND8 mice. Animals were injected with BrdU at 5 weeks of age and examined at selected time points over an eight-week period. Immunocytochemistry analysis of BrdU incorporation indicated that the majority of the BrdU-positive cells were found in the dentate gyrus. The number of BrdU-positive cells was greater in TgCRND8 compared to Non-Tg littermates. Two-way ANOVA revealed significant differences between TgCRND8 and Non-Tg at each time point. Data are means ± S.D. *n* = 5 (^∗^*p* < 0.0001).

BrdU labeling was used to determine whether cell proliferation is impaired or promoted in TgCRND8 mice. In animal injected at 5 weeks (low brain Aβ levels) and the majority of the BrdU-positive newborn cells were seen in the first 2 weeks post-injection. The number of BrdU-positive cells subsequently decreased by half within 4 weeks after BrdU injection (Fig. 1). Additionally, the absolute number of BrdU-positive cell population in the dentate gyrus of TgCRND8 mice was significantly increased as compared to the Non-Tg littermate controls (Fig. 1). The initial increase in the number of BrdU-positive cells in TgCRND8 mice at weeks 1–4 after the BrdU injection (6–9 weeks of age) occurs prior to the increase in Aβ production. The explanation for this observation is unclear but, may relate to the high levels of expression of APP and the presence of high concentrations of soluble neurotrophic APP (sAPPα/β). This explanation is supported by several prior observations including observations that: sAPPα is neurotrophic and promotes survival of cultured neurons (Mattson et al., 1993; Mucke et al., 1996); there is a correlation of *in vivo* sAPPα with improvements of spatial memory that could be due to enhanced neurogenesis (Anderson et al., 1998); and secreted APP can promote proliferation of hippocampal NPCs in culture (Baratchi et al., 2012). It would be interest as part of a future study to examine the effects of γ-secretase inhibitors and determine if decreased levels of sAPPα correlated with any changes in observed increase in the progenitor population.

### Differentiation of labeled progenitors in young TgCRND8 mice

**Fig. 2:**
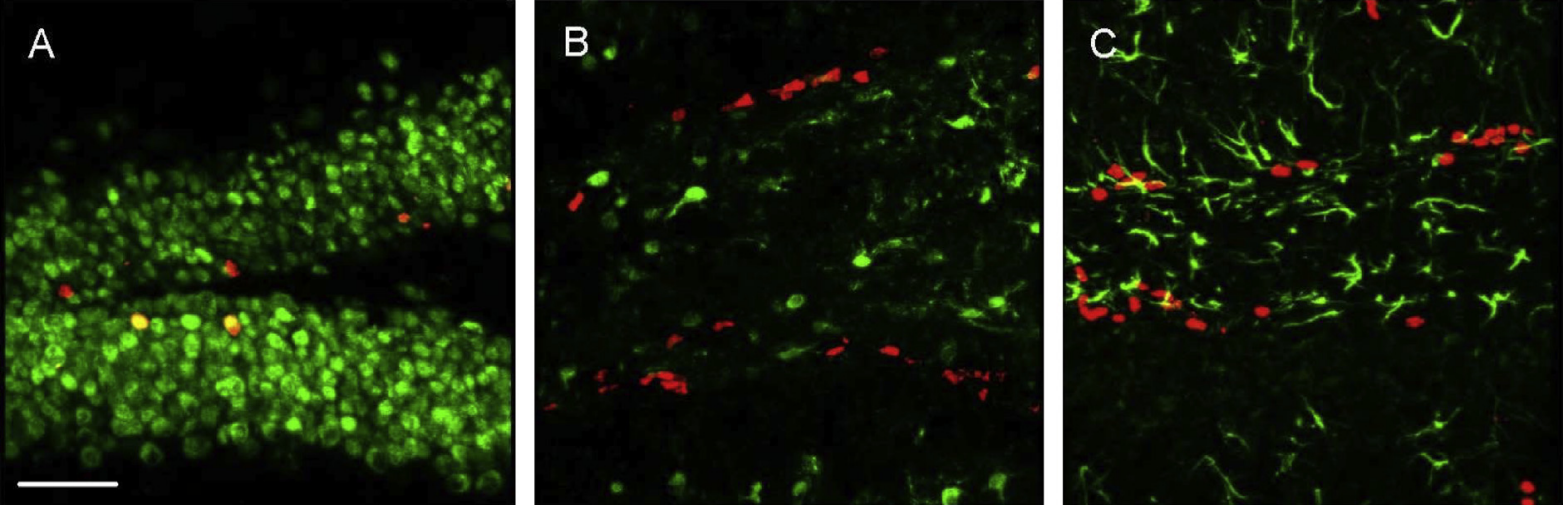
Confocal microscopy images of BrdU with neuronal and glial markers in the dentate gyrus. Representative images are shown for immunostaining conducted in TgCRND8 amyloid mice at 6 weeks of age (1 week post-injection of BrdU). (A) Brain sections were co-labeled with BrdU (red) and a marker of mature neurons NeuN (green), (B) astrocytic marker S100β (green), and (C) astrocytes or Type 1 hippocampal progenitor marker GFAP (green). Scale bar = 100 μm.

To identify the cell types undergoing differentiation in TgCRND8 mice during the period between 5 weeks and 8 weeks of age (i.e. prior to the onset of increased brain Aβ), sections were counterstained with markers for neurons and astrocytes. Counterstaining with neuronal markers revealed that BrdU+/NeuN+ cells were the most predominant class of newly born cells at 1 week post-injection of BrdU in the 5-week-old TgCRND8 mice (Fig. 2A). In contrast, newly generated astrocytes marked by BrdU+/S100β+ (Fig. 2B) or by BrdU+/GFAP+ (Fig. 2C) were less prevalent.

**Fig. 3:**
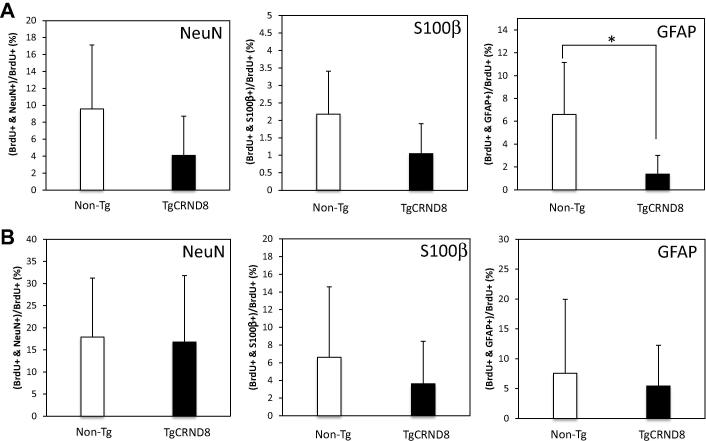
Differentiation of newborn cells in Non-Tg and TgCRND8 mice. BrdU-labeled cells co-expressing mature neural marker NeuN, mature astrocytic marker S100β or astrocyte/Type 1 hippocampal progenitor marker GFAP were examined at different time points in TgCRND8 mice as compared to Non-Tg littermates. (A) One week after BrdU injection in 5-week-old mice, the number of BrdU immunoreactive cells co-labeled with neural/glial markers in the dentate gyrus was counted. (B) Eight weeks after BrdU injection into 5-week-old mice, the number of BrdU-immunoreactive cells co-labeled with neural/glial markers in the dentate gyrus was counted. Data are means ± S.D. *n* = 5 (^∗^*p* < 0.05).

Careful quantification of the BrdU-positive neuronal and astrocytic revealed the surprising observation that although there were more BrdU-positive cells overall in 5-week-old TgCRND8 than in Non-Tg mice, there was a tendency towards a reduction in the number of BrdU+/NeuN+ or BrdU+/S100β+, and more BrdU+ alone cells compared to Non-Tg littermates at both one week and 8 weeks after the BrdU injection (Fig. 3). Because of the high variability in cell counts, this trend only reached statistical significance for BrdU+/GFAP+ double-labeled progenitor cells (Fig. 3A). Nevertheless, these results suggest that even in young TgCRND8 mice the differentiation to NPCs in dentate gyrus is impaired even though the total number of proliferating, BrdU-positive cells was increased.

### Neural progenitor cell viability and Aβ processing *in vitro*

**Fig. 4:**
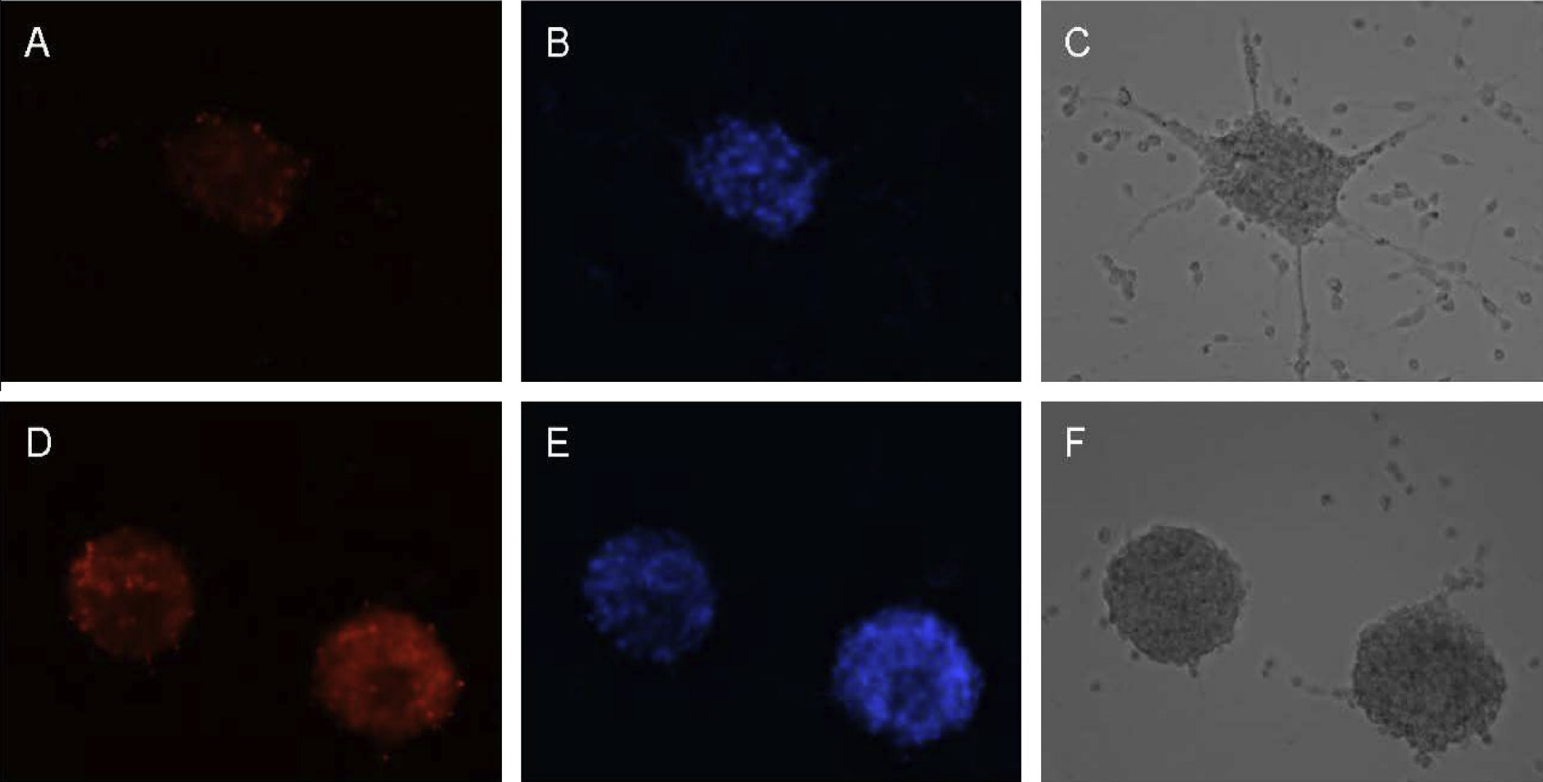
Viability assessments of neurospheres. Cells were isolated from TgCRND8 and non-transgenic littermates at 14 weeks of age and maintained in culture for a period of 4 days. (A) Propidium iodide and (B) Hoechst 33258 staining of Non-Tg mouse neurospheres with (C) visible microscopy. (D) Propidium iodide staining of neurospheres isolated from TgCRND8 mice indicated increased levels of cell death. (E) Hoechst 33258 staining of TgCRND8 neuropheres and (F) visible microscopy.

**Fig. 5:**
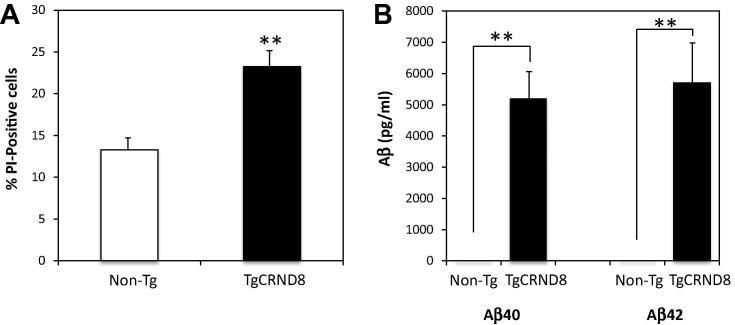
Aβ production and NPCs viability. Neurospheres were isolated from 14-week-old TgCRND8 and non-transgenic littermate and assessed for cell survival and Aβ secretion. (A) Quantification of cell viability based on percentage of propidium iodide (PI)-positive cells in control Non-Tg and TgCRND8 mice. Data are means ± S.D. (^∗∗^*p* < 0.01). (B) Levels of Aβ40 and Aβ42 in NPCs after 4 days in culture as determined by ELISA indicated significant elevations for both peptides as compared to Non-Tg control mice. Data are means ± S.D. (^∗∗^*p *< 0.01).

To determine why the newborn cells in TgCRND8 brain had reduced differentiation into neuronal or glial lines, we isolated neural progenitors from TgCRND8 mice and Non-Tg littermates at 14 weeks of age and examined these progenitor cells for changes in cell viability and Aβ secretion. As a measure of cell death, PI uptake was assessed in cells grown in culture. At 4 days in culture, TgCRND8 neural progenitors showed a significantly higher percentage of PI-positive cells as a ratio of Hoechst nuclear staining (Fig. 4). Quantification of cell viability indicated that the TgCRND8 neurospheres had an ∼15% higher level of cell death based on PI-staining as compared to Non-Tg cells (Fig. 5A). By 7 days of culture, cell death was so extensive in the TgCRND8 neurospheres that cultures were no longer viable and comparisons with the Non-Tg cells were not possible (data not shown).

**Fig. 6:**
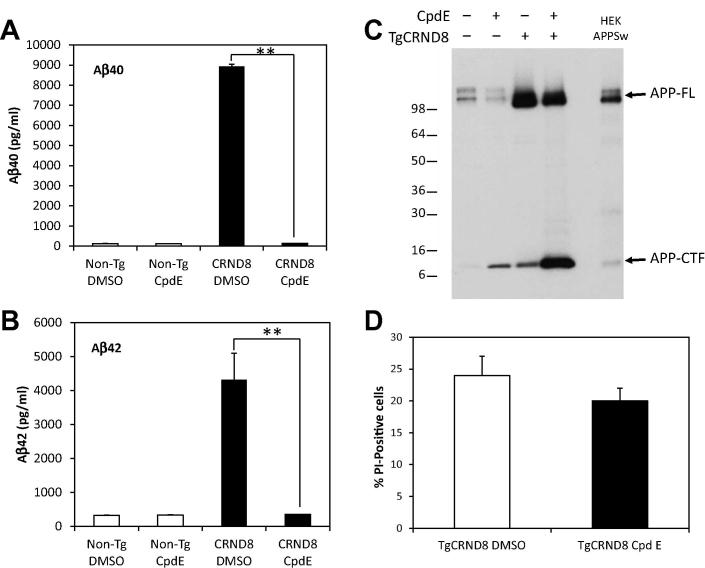
Effects of γ-Secretase Inhibitor on NPCs survival. Neurospheres were isolated from 14 weeks and treated overnight with the γ-secretase inhibitor, Compound E (CpdE), and effects on APP processing and cell viability were assessed. Quantification of (A) Aβ40 and (B) Aβ42 levels by ELISA from conditioned media demonstrated a significant reduction in amyloid processing in the NPCs isolated from TgCRND8 mice as compared to DMSO-treated controls. Data are means ± S.D. (^∗∗^*p* < 0.01). (C) Western blotting for APP confirmed the high level of full-length APP (APP-FL) expression in the TgCRND8 mice as compared to Non-Tg animals and increased C-terminal fragment (APP-CTF) following γ-secretase inhibitor treatment. (D) Survival of the TgCRND8-dervied NPCs was only modestly reduced by γ-secretase inhibition as determined by propidium iodine staining. Data are means ± S.D.

To determine whether the accelerated mortality of the neural progenitors might arise from the secretion of neurotoxic Aβ42 into the medium, we used a standard ELISA to assess Aβ levels in media from Non-Tg and TgCRND8 cultures. The media from control cultures showed little or no Aβ40 or Aβ42 (Fig. 5B). In contrast, the conditioned media from the TgCRND8-derived neural progenitors contained high levels (⩾5000 pg/ml) of both Aβ40 and Aβ42 (Fig. 5B). The possibility of a direct Aβ toxicity on the TgCRND8-derived NPCs was investigated further using the γ-secretase inhibitor, Compound E. Treatment of the cells with this inhibitor over the course of 12–14 h virtually eliminated the production of human Aβ40 and Aβ42 which were reduced to levels comparable to Non-Tg cells (Fig. 6A, B). Immunoblotting of cell lysates confirmed the high expression of full-length APP in the TgCRND8 mice as compared to Non-Tg mice as well as cells stably expressing the APPSw mutant (Fig. 6C). In addition, treatment with Compound E resulted in a substantial increase in the APP C-terminal fragment of NPCs isolated from the TgCRND8 mice (Fig. 6C). A detectable increase in the APP-CTF from Non-Tg cells was also observed which is consistent with the γ-secretase inhibition. Under these conditions, viability of the TgCRND8 progenitor cells was only modestly decreased suggesting that decreased Aβ secretion may be only one of the contributing factors in the observed NPC toxicity (Fig. 6D). However, this may be complicated by the inherent toxicity of the APP-CTF as has been shown in several previous instances (Song et al., 1998; Berger-Sweeney et al., 1999). The cytotoxic properties of the APP-CTF (or C99) were recently reported in the triple transgenic mouse model where elevations of this fragment resulted in extensive neurodegeneration prior to Aβ deposition (Lauritzen et al., 2012). In this case, pharmacological intervention with a comparable γ-secretase inhibitor (ELN006) resulted in significant cell toxicity in the absence of Aβ secretion similar to that observed for Compound E-treated NPCs extracted from the TgCRND8 mice. Therefore inhibition of the γ-secretase complex and the subsequent accumulation of the APP-CTF may contribute to the observed cell loss and mask the effects of any Aβ cytotoxicity under these conditions. To determine the extent of amyloid-mediated toxicity, it may therefore be necessary to target Aβ by different approaches such as antibody-mediated removal in cell culture or prophylactic immunotherapy of TgCRND8 animals to examine if there are any changes in NPC survival. However, the findings from our investigation suggest that high levels of APP and Aβ result in a significantly decreased viability of neuroprogenitor cells.

## Conclusion

In the present study, we have shown the increase of proliferation of newborn cells in the dentate gyrus of the hippocampus in young TgCRND8 mice compared with Non-Tg control mice, in contrast, the differentiation to NPCs is impaired. Additionally, neurosphere analyses revealed, cultures isolated from TgCRND8 mice have low viability and high secreted Aβ peptide levels. This significant increase in Aβ peptides secreted by progenitor cells expressing mutant human APP transgenes may contribute to the cytotoxicity associated with the TgCRND8 neurospheres, and may be at least partially why it is not possible to maintain them in culture for extended periods of time. These findings suggest that Aβ, possibly as oligomeric species, plays a key role in the lack of viability of progenitors *in vivo* and *in vitro*. However, contributions from the APP holoprotein or other APP cleavage fragments such as amyloid precursor protein intracellular domain (AICD) cannot presently be fully excluded. Additional experiments will be needed using chimeric (mutant/wild-type) neurosphere cultures and chimeric transgenic animals to determine whether the observed defects in neurogenesis arise from the toxic effects. Aβ is produced by the progenitor cells themselves (i.e. cell autonomous toxicity). If the toxicity is not cell autonomous and if wild-type NPCs are also directly compromised in the presence of high levels of Aβ40 and Aβ42, it will have significant implications for cell-based therapy of AD.

The cumulative evidence from the current study is consistent with a pivotal role for Aβ toxicity and possibly contributions from the APP-CTF in the impairment of proliferation and survival of NPCs in TgCRND8 mice. A similar process may be occurring in early-onset forms of familial AD arising from missense mutant APP, duplicated APP or Trisomy21 suggesting a potential double jeopardy of amyloid-induced neuronal loss coupled with decreased repair abilities and/or decreased inclusion of new neurons in new memory circuits arising from APP/Aβ-mediated defects in the neurogenesis pathways. Indeed, impaired neurogenesis in hippocampal CA3 regions is associated with impaired spatial memory (Niibori et al., 2012) and both the hippocampus and spatial memory are affected in AD.

## Funding

This work was supported by grants from the Canadian Institutes of Health Research (MOP-115056 to P.E.F.); the Firefly Foundation; Krembil Foundation; and Alzheimer Society of Ontario. P.S. G-H is supported by grants from the Wellcome Trust; Medical Research Council; and the National Institutes of Health.
